# New Evidences of Mitochondrial DNA Heteroplasmy by Putative Paternal Leakage between the Rock Partridge (*Alectoris graeca*) and the Chukar Partridge (*Alectoris chukar*)

**DOI:** 10.1371/journal.pone.0170507

**Published:** 2017-01-23

**Authors:** Andrea Gandolfi, Barbara Crestanello, Anna Fagotti, Francesca Simoncelli, Stefania Chiesa, Matteo Girardi, Eleonora Giovagnoli, Carla Marangoni, Ines Di Rosa, Livia Lucentini

**Affiliations:** 1 Department of Biodiversity and Molecular Ecology, Research and Innovation Centre, Fondazione Edmund Mach (FEM), San Michele all’Adige, Trento, Italy; 2 Department of Chemistry, Biology and Biotechnologies, University of Perugia, Perugia, Italy; 3 Department of Biology & CESAM, University of Aveiro, Aveiro, Portugal; 4 Civic Museum of Zoology, Rome, Italy; University of Innsbruck, AUSTRIA

## Abstract

The rock partridge, *Alectoris graeca*, is a polytypic species declining in Italy mostly due to anthropogenic causes, including the massive releases of the closely related allochthonous chukar partridge *Alectoris chukar* which produced the formation of hybrids. Molecular approaches are fundamental for the identification of evolutionary units in the perspective of conservation and management, and to correctly select individuals to be used in restocking campaigns. We analyzed a Cytochrome oxidase I (*COI*) fragment of contemporary and historical *A*. *graeca* and *A*. *chukar* samples, using duplicated analyses to confirm results and nuclear DNA microsatellites to exclude possible sample cross-contamination. In two contemporary specimens of *A*. *graeca*, collected from an anthropogenic hybrid zone, we found evidence of the presence of mtDNA heteroplasmy possibly associated to paternal leakage and suggesting hybridization with captive-bred exotic *A*. *chukar*. These results underline significant limitations in the reliability of mtDNA barcoding-based species identification and could have relevant evolutionary and ecological implications that should be accounted for when interpreting data aimed to support conservation actions.

## Introduction

The rock partridge *Alectoris graeca*, Meisner 1804, is a polytypic species included in “The IUCN (International Union for Conservation of Nature) Red List of Threatened Species” as “Near Threatened” on a global scale [1] and as “Vulnerable” in Italy [2], due to a trend of population decline. This decline is mostly associated with anthropogenic causes, including the massive release of captive-bred chukar partridge *Alectoris chukar*, Gray 1830, resulting in the formation of hybrids [3, 4, 5]. Genetic pollution and hybridization magnified the damages already caused by the decline of natural populations due to habitat loss and overhunting, and likely resulted from an uncertain morphological distinction between these species. This difficulty is almost entirely due to non-discrete characters, not easily applicable [6] and even less effective to detect hybrid individuals.

In recent years, molecular markers have been proved to represent an effective tool to support management of *Alectoris* populations [3, 4, 5, 7, 8]. As in most conservation-oriented genetic studies, both mitochondrial DNA (mtDNA) and nuclear DNA (nDNA) were used [3, 4, 5, 7], offering different and complementary information on the history of populations. While biparentally inherited nDNA markers can trace both the paternal and maternal contribution along generations, maternally inherited mtDNA is usually informative of matrilinear lineages only. The classical view of unilinear mode of inheritance, with no recombination, together with a relatively high mutation rate made mtDNA particularly appealing as a molecular tool in evolutionary and conservation biology, offering an informative and simpler system to model population history compared with nDNA [9]. However, the general assumption of uniparentally transmitted, homoplasmic and non-recombining mitochondrial genomes is more and more contrasted by accumulating evidences of exceptions to the rule, with a growing list of taxa in which mtDNA biparental inheritance, heteroplasmy and recombination were demonstrated (reviewed in [10, 11, 12]).

Heteroplasmy has been observed in a range of eukaryotic taxa, in plants, fungi and animals [10]. While point heteroplasmy, i.e. the within individual co-occurrence of different mtDNA haplotypes differing from each other at a single (or few) nucleotide position(s), can be a consequence of somatic or germline mutations, heteroplasmy can alternatively result through mitochondrial paternal leakage [13]. Early studies detected heteroplasmy associated to paternal leakage substantially only in hybrid zones [10]. Nevertheless, recent studies [14, 15] demonstrated that heteroplasmy can be common within single populations as well. In fact, on the one hand, when leakage occurs either between closely related populations, with no mitochondrial structure, or within single populations, with no mitochondrial variation, paternal mtDNA may simply not be detectable due to high similarity or identity between haplotypes [9]; on the other hand, the selective elimination of sperm mitochondria might not function properly when mtDNA divergence is elevated between parents [10, 16]. Studies of natural or anthropogenic hybrid zones, in which relatively different mitochondrial haplotypes come into contact, can thus provide an ideal experimental system to search for the evidence of paternal mitochondrial leakage [13].

Here we report on the observation of putative introgression-associated paternal leakage of mitochondrial haplotypes in two individuals from an anthropogenic hybrid zone of *A*. *graeca* and introduced *A*. *chukar*. We discuss the evolutionary and conservation implications of the observed evidence to our knowledge described here for the first time, in the endangered taxon of Galliformes, and their potential relevance in a conservation context.

## Materials and Methods

### Ethics statement

The work performed during the analyses carried out for this manuscript is consistent with the National regulations and indications of the Ethics Committee of the University of Perugia (Italy). Approval by Ethics Committee was not necessary given the nature of the data collected (museum specimens, feathers) and the method of data recovery, without any animal suffering.

For museum specimens, each museum Direction agreed with this sampling campaign. Authorization of the CAMS’s Director and of the Morbegno’s museum were reported (S1 File). No specimen was sampled in absence of the museum’s staff.

### Molecular markers

A central, taxonomically informative, fragment of the mtDNA Cytochrome oxidase c subunit 1 (*COI*) in both putative *A*. *graeca* and *A*. *chukar* specimens was characterized. Investigations were performed on 44 either contemporary or historical samples of *A*. *graeca* and *A*. *chukar* (Table 1).

**Table 1:** *A*. *graeca* and *A*. *chukar* analysed samples. Details of analyzed specimens for each species: contemporary or historical samples, sample date, sampling location and geographic area of origin, number of analyzed samples (N) and Catalog number (for museum specimens).

***A*. *chukar***					
	**Catalog number**	**Sample date**	**Origin**	**N**	**Area**
**Contemporary**		live	Torre Certalda	7	Central Italy (Umbria)
		2014	Macedonia	1	Republic of Macedonia
		2015	Cardosa Mountain	1	Sibillini's Park—Central Italy
		2015	Fema Mountain	8	Sibillini's Park—Central Italy
	505, 743, 205	1952–1995	Morbegno's Natural History Museum	3	Northern Italy
***A*. *graeca***					
	**Catalog number**	**Sample date**	**Origin**	**N**	**Area**
**Historical**	Unique sample.	II half '800	Città della Pieve Museum	1	Central Italy
	Private collection	1905	Abruzzo Region	1	Central Italy
	Private collection	1905	Maggio Mountain—Umbria Region	1	Central Italy
	AV703, AV705	exact date not available	Calci's (Pisa) Natural History museum	2	Central Italy
	2545	1908	Zoological Museum of Rome City	1	Northern Italy
	casal2, casal4	1800	Casalina's Natural History Gallery	2	Central and Southern Italy
**Contemporary**		2015	Cardosa Mountain	5	Sibillini's Park—Central Italy
		2015	Fema Mountain	7	Sibillini's Park—Central Italy
	34, 518, 843	1955–1995	Morbegno's Natural History Museum	3	Northern Italy
	2527	1956	Zoological Museum of Rome City	1	Northern Italy

For *A*. *graeca*, specimens were classified as “contemporary” if either alive or taxidermied after 1930, the date of the first documented introduction for chukar partridge in Italy [17]. Museum specimens (“historical”), collected before the onset of stocking practices, may in facts represent the key to the recognition of *A*. *graeca* genetic makeup, thus avoiding misinterpretations. Both alive and museum specimens were morphologically assigned to either of the two species [6] and were sampled with a non-invasive method, collecting few feathers (from three to five).

DNA was extracted from a single feather [18] for each specimen in duplicate and subsequent PCR amplification and sequencing were performed according to previous published protocols [18, 19]. In particular, a first amplification with Bird-F1 and Bird-R1 primers [20] was done followed by a nested amplification with primers 5-NEST-F (GTAATCGTTACAGCCCATGC) and 3-NEST-R (gggtcgaaaaatgtggtgtt). All reactions were run using the following thermal cycle program: 5’ at 94°C followed by twenty-six cycles of 30” at 94°C, 45” at 52°C, and 45” at 72°C, followed by a final extension of 1’ at 72°C. The risk to amplify nuclear insertions of mtDNAs (nuclear mitochondrial DNA, NUMTs) was taken into account and prevented by the use of feathers as DNA source [21] and by a careful design of the *COI* nested primers [22]. Specific features of NUMTs, possibly useful for their recognition [21, 23], were finally evaluated on the obtained sequences. PCR products were run on 2.5% agarose gel clearly showing a single band, with no evidence of smaller amplicons. Samples of different species were processed in different laboratories and by using dedicated laminar flow hoods to avoid cross-contamination between contemporary/museum samples.

To exclude sample contamination, a subset of seven individuals was also genotyped with ten microsatellite loci [13]: six from the “ARU” series [24] and four from “MCW” series [4, 25]. The ten loci were analyzed following authors’ protocols [24, 26].

## Results and Discussion

**Fig 1:**
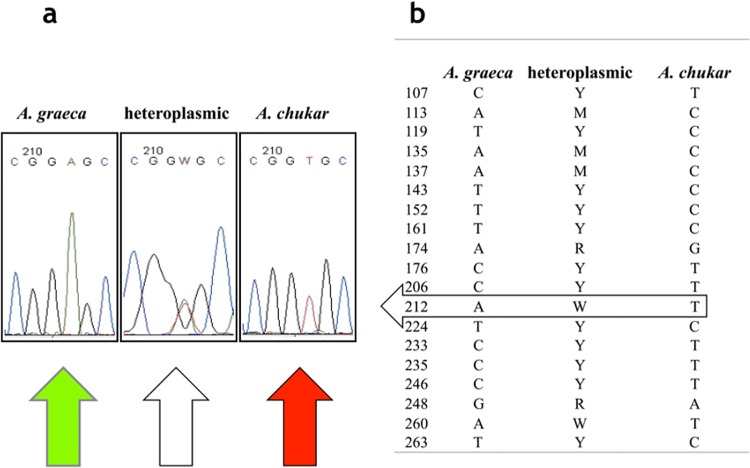
*COI* heteroplasmy in *Alectoris* samples. (a) Example of polymorphic site at position 212, clearly showing mtDNA *COI* heteroplasmy. (b) Heteroplasmic sites (GenBank KT180225) compared with polymorphisms discriminant in *A*. *graeca* (GenBank KT180224)*/ A*. *chukar* (GenBank KT180226) attribution.

Sequences of 312 bp were aligned and screened for polymorphism, and the different haplotypes retrieved were deposited in GenBank (Accession Numbers KT180220-KT180226). Out of 312 bp, 41 sites were polymorphic in the alignment. Morphology based assignment of individuals to species was univocally confirmed in all cases but two. In two contemporary samples from Monte Fema (see Table 1) morphologically attributed to *A*. *graeca*, 19 polymorphic sites, all confirmed from duplicated independent analyses for each sample, clearly showed heteroplasmy (Fig 1a). The observed pattern of polymorphism suggested these two specimens to be derived from hybridization between *A*. *graeca* and *A*. *chukar* (see Fig 1b).

The presence of NUMTs can be reasonably excluded in the present study. First, NUMTs are usually shorter than the corresponding mitochondrial gene [23]. In the *Alectoris* genus NUMTs were described in *A*. *chukar*, where a 351bp NUMT was co-amplified with a 1143 bp-*Cytb* fragment (see Table 4 in [23], for a review of base count of mitochondrial genes vs NUMTs in ten Galliformes). Furthermore, NUMTs are usually recognizable *ex post* through analysis of reading frame and mutation positions. For example, in *Gallus gallus* identities between NUMTs and mitochondrial sequences are variable, ranging from 58.6 to 88.8% [27]. Specifically, no amino acid variation, no stop codon insertion, nor sequence length variation, which can be found in NUMTS of coding regions [21], were observed in the present work.

The two haplotypes observed in each of the two individuals were easily reconstructed by phasing polymorphic sites through comparison with the other haplotypes observed in the sample set. The two resulting haplotypes, identical in the two individuals (GenBank KT180225), are too differentiated to be resulting from somatic mutations; at the same time, they are strictly related to one of the two species’ mitochondrial haplogroups—without presenting frame shifts or early stop codon mutations—to represent nuclear copies of the gene. In fact, the same sequences of the two haplotypes were observed in 5 individuals of *A*. *graeca* (GenBank KT180224, from Umbria and Abruzzo Regions, Central Italy) and in all the individuals of *A*. *chukar* (GenBank KT180226, Fig 1b).

In order to exclude that the observed heteroplasmic sequences might be due to sample contamination, ten microsatellite loci were screened on a subset of seven individuals, including the two heteroplasmic ones. The scored microsatellite allelic ranges were compatible with those already reported for the genus *Alectoris*, as showed in Table 2.

**Table 2:** Summary data of the microsatellites used in the present study. Specific locus, fluorochrome used, alleles range and number of alleles observed in the present work.

Locus	Labelling fluorochrome	Allele range	Number of alleles
**ARU1.9**	FAM	126–156	7
**ARU1.19**	FAM	190–194	2
**ARU1.22**	PET	92–98	2
**ARU1.23**	VIC	179–185	4
**ARU1.27**	PET	202–208	4
**ARU1.29**	NED	158–180	8
**MCW135**	VIC	112–114	2
**MCW225**	PET	152–168	5
**MCW276**	NED	207–211	2
**MCW295**	NED	84–90	4

In both the heteroplasmic specimens, as well as in the other screened individuals, a clear homozygote or heterozygote biallelic signal was observed, referable to a single diploid individual, thus strongly rejecting the hypothesis of cross-sample contamination.

To our knowledge, this is the first record of heteroplasmy in *A*. *graeca* and the first record of a likely mitochondrial paternal leakage in Galliformes. In fact, the only case of mtDNA heteroplasmy from the literature for a congeneric was observed in the cytochrome b sequence of an *A*. *chukar* specimen from Cyprus [28]. The two haplotypes observed in the heteroplasmic individual (Cyp_27_), both included in the *A*. *chukar* haplogroup and differing by a single nucleotide polymorphism, were shared by one (AM084572) and 15 (AM084573) different samples, respectively, in the same dataset. While the authors did not discuss the possible origin of the observed heteroplasmy, this could however represent another case of paternal leakage, occurred within species. Besides confirming our observation, this interpretation of the data [29] would also suggest that paternal leakage might be not so infrequent in this taxon. This would in turn have relevant evolutionary and ecological implications that should be accounted for when interpreting data generated in order to support conservation actions. As an example, in a survey of introgression patterns between rock and chukar partridge [4] no evidence of maternal introgression from the exotic species, i.e. the presence of chukar mtDNA haplotypes, was observed in 28 putative hybrid individuals determined by microsatellite analysis. The authors therefore concluded that captive-bred female partridges reproduced poorly in nature and introgression was therefore unidirectional, by backcrossing with native wild female rock partridges [4]. This deduction might be reconsidered in the light of possible paternal leakage. Mitochondrial population bottlenecks (intra-individual genetic drift or vegetative sorting [29]), during the gametogenesis cell divisions, are supposed to quickly eliminate heteroplasmy, in a single or few generations [10, 30]. The expected unbalanced and relatively limited proportion of paternal mtDNA to maternal mtDNA makes the former more prone to loss by drift [9]. However, heteroplasmy may persist through generations and become fixed, or alternatively maternal or paternal mtDNA may be lost due to either the forces of drift or selection [9]. Thus, it’s possible that paternal leakage-associated hybridization might contribute to the mechanism through which genetically divergent mitochondrial genomes can meet and recombine, purging deleterious mutations and promoting the generation of new mitochondrial haplotypes, possibly contributing to increased fitness [10, 31]. Although Robison and colleagues [15] work on a parasite, having obviously a complete different biology than *Alectoris*, it is interesting what they argue for *C*. *lectularius*, the bed bugs. They hypothesize that paternal leakage may be really common and that its effects may be considered “long lasting”. Furthermore, they suggest a fascinating hypothesis circa the correlation between the insurgence of mitochondrial heteroplasmy and the re-union of historically allopatric lineages, that may simulate “hybridization-like” events [15]. In the two *Alectoris* species, maybe a reproductive statement occurs similar to that hypothesized for allopatric lineages in which the molecular mechanisms preventing “transmission of paternal mtDNA to the oocyte may have been relaxed, leading to extensive paternal mtDNA leakage in inter-population hybrids” [15]. This hypothesis should be evaluated in the future also in the light of the evolutive history of the *Alectoris* genus.

## Conclusions

This paper documented, for the first time, the presence of mtDNA heteroplasmy in *A*. *graeca / A*. *chukar*. The observed heteroplasmy is reasonably associated with paternal leakage, following hybridization between the two species. This evidence confirms that paternal leakage should be taken into account in order to avoid erroneous reconstruction of population or species histories in mtDNA analyses, and—if frequent enough—might have possible far-reaching effects on the evolution and persistence of populations and species [32], acquiring relevant and fundamental importance for conservation purposes.

## Supporting Information

S1 File Authorizations to draw museums’ specimens. The Morbegno’s museum (first page) and the CAMS’s (second page) Directors authorizations to draw museums’ specimens are reported.
(PDF)
