# Supplementary material for: New Evidences of Mitochondrial DNA Heteroplasmy by Putative Paternal Leakage between the Rock Partridge (Alectoris graeca) and the Chukar Partridge (Alectoris chukar)
Source: PLoS One. 2017 Jan 23;12(1):e0170507. doi: 10.1371/journal.pone.0170507 (PMC5256862; doi:10.1371/journal.pone.0170507)
Supplement: S1 File — The Morbegno’s museum (first page) and the CAMS’s (second page) Directors authorizations to draw museums’ specimens are reported. (PDF) [file pone.0170507.s001.pdf]

S1 File. Authorizations to draw museums' specimens. The Morbegno's museum (first page) and the CAMS's (second page) Directors authorizations to draw museums' specimens are reported.

Museo civico di Storia naturale
Via Cortivacci, 2
23017 Morbegno
tel. e fax 0342 612451
museo@morbegno.it
www.morbegno.it/museo

http://www.morbegno.it/museo

MORBEGNO
museo
civico
di storia
naturale

dott.ssa Livia Lucentini,
Dipartimento di Chimica,
Biologia e Biotecnologie
Università degli Studi di Perugia
Via Pascoli
06123 Perugia

Morbegno, 18/03/2015
Prot. n.

OGGETTO: **Invio campioni genere *Alectoris* per analisi genetica**

Come da sua richiesta del 4/03/2015 invio per la vostra ricerca i campioni degli esemplari del genere *Alectoris* della collezione di Uccelli di questo Museo con n. di inventario: 33-34-36-37-74-75-203-205-415-505-518-622-743-748-843 di cui ho già spedito via mail foto ed estratto del catalogo.

Rimango in attesa di ricevere, come da lei indicato, l'esito delle analisi genetiche da allegare ai dati di ciascun esemplare.

Ringrazio e porgo cordiali [illegible]

UNIVERSITA' DEGLI STUDI DI PERUGIA
CENTRO DI ATENEO PER I MUSEI SCIENTIFICI - C. A. M . S.
Il Direttore: Prof.ssa Cristina Galassi
cristina.galassi@unipg.it

CAMS

Perugia, 25 marzo 2015
Prot. N. 139/2015

Alla c.a. della dott.ssa Livia Lucentini
Dipartimento di Chimica, Biologia e Biotecnologie dell'Università degli Studi di Perugia
SEDE

Oggetto: *richiesta prelievo di campioni del piumaggio di* Alectoris graeca *e* Perdix perdix *conservati presso la Galleria di Storia Naturale dell'Università degli Studi di Perugia in località Casalina di Deruta – PG.*

Gentile dott.ssa Livia Lucentini,
in riferimento a Sua richiesta del 12.03.2015, Le comunico che autorizzo il prelievo di parti del piumaggio delle specie in oggetto alle seguenti condizioni:

- Il prelievo, del tutto conservativo, dovrà essere effettuato da Lei insieme con il personale della Galleria di Storia Naturale (Sergio Gentili e Angelo Barili);
- Le eventuali immagini e/o i relativi dati storici rilevabili da catalogo e/o dal cartellino, degli esemplari, dovranno essere ottenute/i sotto la supervisione del personale della Galleria (Sergio Gentili e Angelo Barili);
- I dati scientifici che deriveranno dalle ricerche che hanno avuto per oggetto i campioni prelevati presso le collezioni della Galleria di Storia Naturale, una volta pubblicati, dovranno poter essere utilizzati anche dalla Galleria di Storia Naturale per le eventuali attività di conservazione, documentazione e valorizzazione degli esemplari da cui sono stati prelevati
- qualunque produzione scientifica riguardante i campioni prelevati (tesi di laurea, report, articoli su riviste scientifiche nazionali ed internazionali,…) dovrà riportare chiaramente l'origine museale dei campioni citando anche il CAMS.

Riguardo all'organizzazione logistica delle attività elencate, La prego di contattare Sergio Gentili (sergio.gentili@unipg.it – 0759711077) in modo da verificare la disponibilità dei campioni e concordare insieme date ed orari.

Cordiali saluti.

UNIVERSITA' DEGLI STUDI DI PERUGIA – CENTRO DI ATENEO PER I MUSEI SCIENTIFICI

Il Direttore del CAMS
(Prof.ssa Cristina Galassi)
